# Nitrous Oxide as an Anæsthetic

**Published:** 1863-11

**Authors:** 


### NITROUS OXIDE AS AN ANÆSTHETIC.

The attention of the profession has been directed within the last few months, to this agent for the production of anæsthesia. It is claimed by those who have experimented with it, that it is very efficient, operating rapidly, almost as much so as chloroform, and with perfect safety; no serious accident yet having occurred from its use. Patients are as easily controled while under its influence as any other anæsthetic agent.

The manner of its action we conceive to be very different from that of chloroform or ether; during the administration of the latter, the tendency is to deprive the blood of the ordinary amount of oxygen, while the former increases it. While death even *might* ensue from asphyxia under its full influence, yet its operation upon the nervous system, and especially upon the nerve centres, is certainly attended with far less danger than chloroform or ether.

The manner of administering it seems to constitute the greatest objection to its general use. With appropriate apparatus it is easily prepared, but it requires large space; three to six gallons being required for each case. The apparatus and mode of administration are being simplified, and it is presumable that this will be so far accomplished, that there will be no special objection in this direction. All the apparatus, with instructions for using it, is furnished by parties in New York, and other points. We hope all who have opportunity will experiment and report.

